# Unveiling the Gray: A Rare Case of Gray Platelet Syndrome With Hepatomegaly and Immune Dysregulation in a 14-Year-Old

**DOI:** 10.1155/crh/3253411

**Published:** 2025-10-14

**Authors:** Muhammad Takhman, Moath Hattab, Reem Shihab, Asmaa Sarama, Sultan Mosleh

**Affiliations:** ^1^Department of Medicine, Faculty of Medicine and Allied Medical Sciences, An-Najah National University, Nablus, State of Palestine; ^2^Division of Hematology/Oncology, Department of Medicine, An-Najah National University Hospital, Nablus, State of Palestine

## Abstract

Gray platelet syndrome (GPS) is a rare inherited platelet disorder characterized by the presence of gray platelets on blood smears, resulting from a deficiency of *α*-granules. The thrombocytopenia presents in a spectrum of bleeding tendencies, varying among different patients. We present a case of a 14-year-old male presenting with recurrent epistaxis, thrombocytopenia, hepatosplenomegaly, and recurrent infections that had not been diagnosed previously. Whole-exome gene sequencing revealed a homozygous likely pathogenic splice-site variant in the NBEAL2 gene, confirming the diagnosis of GPS, which is inherited in an autosomal recessive manner due to biallelic variants in NBEAL2. The patient had atypical hepatomegaly and low lymphocyte and monocyte counts, findings consistent with emerging evidence that GPS affects multiple hematopoietic lineages. It also contributes to immune dysregulation and results in increased susceptibility to autoimmune disorders, highlighting the need for guidelines to screen for autoimmune complications in GPS patients.

## 1. Introduction

Gray platelet syndrome (GPS) is a rare form of inherited platelet disorder (IPD) that is characterized by macrothrombocytopenia and gray platelets on Wright-stained blood smears due to a lack of *α*-granules and their contents. It was first described by Raccuglia in 1971 [1]. It is inherited in an autosomal recessive pattern. Its diagnosis is made by electron microscopy (EM), which reveals the absence or marked reduction of *α*-granules in platelets. These granules serve as the primary storage site for hemostatic proteins and glycoproteins involved in inflammation and wound healing [2].

The underlying genetic cause of GPS is biallelic variants in the NBEAL2 gene, located on Chromosome 3 (3p21), which encodes the Neurobeachin-like 2 (NBEAL2) protein. NBEAL2 is a member of the BEige and Chediak–Higashi (BEACH) domain–containing protein family and is believed to play a critical role in vesicular trafficking and the development of *α*-granules [3].

Patients with GPS typically present in infancy or early adolescence with an increased bleeding tendency of variable severity. Clinical manifestations may include epistaxis, easy bruising, postoperative hemorrhage, and, in some cases, intracranial hemorrhage [4]. Myelofibrosis and splenomegaly may also occur, although life expectancy is generally normal. Management is mainly supportive and includes platelet transfusions before surgical procedures and administration of 1-desamino-8-D-arginine vasopressin (DDAVP) [2].

Due to its rarity and variable presentations, GPS remains a challenging diagnosis. This report describes a 14-year-old male patient who presented with multiple nonspecific symptoms but had no definitive diagnosis until a biallelic likely pathogenic variant in NBEAL2 was identified via genetic analysis.

## 2. Case Presentation

A 14-year-old male presented with a history of hepatosplenomegaly, thrombocytopenia, recurrent epistaxis, recurrent chest infections, and recurrent episodes of prolonged high-grade fever up to 41°C. Despite previous evaluations, no definitive diagnosis was established. He was referred to our hospital to rule out hemophagocytic lymphohistiocytosis (HLH). The patient also had a positive family history of similar complaints in three relatives. Notably, there was no history of other bleeding episodes from any site, no skin rashes, or bruising.

On physical examination, the patient was hemodynamically stable with normal vital signs. Abdominal examination revealed a slightly distended abdomen, with the spleen palpable 2 cm below the costal margin and the liver palpable 2 cm below the costal margin.

Initial laboratory tests revealed mild leukopenia, thrombocytopenia, slightly prolonged prothrombin time (PT), and slightly elevated international normalized ratio (INR) Table 1. Abdominal ultrasonography revealed a liver measuring 15 cm in craniocaudal dimensions, with a normal outline and echogenicity, and no definite focal lesions. The spleen measured 14 cm in the long axis and appeared heterogeneous.

The patient was initially managed with maintenance intravenous fluids and allopurinol as prophylaxis for tumor lysis syndrome (TLS). He did not receive chemotherapy, and there was no oozing or bleeding from any site. Bone marrow aspiration and trephine biopsy were performed without complications, and the samples were sent for histopathological analysis. The patient was discharged.

Two days after discharge, the patient was readmitted to the pediatric ward due to recurrent vomiting and intolerance of oral intake. On admission, he appeared unwell and dehydrated, with sunken eyes and delayed capillary refill (3 s). He was given 500 mL of intravenous 0.9% normal saline and 8 mg of intravenous ondansetron as a stat dose. He was then placed on maintenance intravenous fluids supplemented with potassium chloride (KCl). By the following day, the patient tolerated oral intake well, with no further episodes of vomiting or diarrhea, and his condition improved significantly. The patient was discharged.

Microscopic examination of the blood smear showed no pathological findings. Histopathological analysis of the bone marrow showed normocellular trilineage hematopoiesis, no morphologic evidence of malignancy, and no increase in stromal histiocytes or emperipolesis.

Whole-exome sequencing (WES) subsequently revealed a homozygous likely pathogenic splice-site variant (c.7225-1G > C) in the NBEAL2 gene, confirming the diagnosis of GPS. This variant has been previously reported in the literature [5] and is listed in ClinVar as likely pathogenic (Variation ID: 804437) [6].

## 3. Discussion

Although GPS is an extremely rare disease, some simple precautions can be used to differentiate it from similar presenting disorders such as immune thrombocytopenia (ITP), HLH, Bernard–Soulier syndrome, myeloproliferative disorders (MPDs), and Von Willebrand disease (VWD).  Table 2 shows the key features to aid in the differentiation between these disorders, as they share common symptoms, most notably bleeding tendencies, which are less common in HLH and MPD. Moreover, they all typically have thrombocytopenia, except for MPD (usually thrombocytosis, but thrombocytopenia may occur in advanced stages) and VWD (normal platelet count, bleeding due to defective platelet adhesion). As GPS might meet the diagnostic criteria for one of these diseases, we place an immense emphasis on viewing the peripheral blood smear on the microscope for any patient with an abnormal complete blood count (CBC) test, as it is a relatively cheap and readily available test. If it shows large platelets with a complete lack of granulation, it can lead to a diagnosis of GPS, and away from other diseases, further confirmation by EM showing the absence of a-granules is required for definitive diagnosis [3].

We suspect the possibility that the blood smear might have not been thoroughly inspected, as most cases of GPS can be identified on blood smears, but the smear was disposed of at the time of writing the case report.

Our patient presented with thrombocytopenia, splenomegaly, and bleeding tendencies, specifically nosebleeds, consistent with other cases in the literature, and hepatomegaly, although it is less frequently highlighted in GPS, it has been previously reported, notably, by a case reported by Steinberg-Shemer and Tamary [5].

A novel study by Sims et al. was conducted on 47 GPS patients and highlighted that NBEAL2 mutations do not only effect platelets but also neutrophils, monocytes, and CD4^+^ T cells, suspecting that it has a vital role in granule formation in many blood cell precursors; in our case, we found a different pattern, where the absolute count of lymphocytes and monocytes were low, in addition to the low the WBC count [3, 15].

GPS affects neutrophils by depleting gelatinase granules and neutrophil-specific granules, reducing NETosis, impairing extracellular trap formation and bacterial defense, and neutrophils are frequently engulfed by megakaryocytes in GPS (emperipolesis), which subsequently results in neutrophilic proteins (ELANE and, MPO) being present in platelets. Monocytes are also typically affected through reduced granule protein abundance, resulting in decreased count and granularity of monocytes. Lymphocytes are also affected by reduced number and less functional natural killer cell, leading to the increased susceptibility to infections; dysregulation of T cell activation and function is suggested to be the contributor to the immune abnormalities and the association with autoimmune disorders; CD4 lymphocytes also showed a positive correlation between gene expression and protein abundance with upregulated inflammatory genes and proteins, indicating heightened proinflammatory activity [15, 16].

Patients with GPS exhibited a much higher prevalence of autoimmune disorders (26%) than the normal population (3%), most commonly Hashimoto's thyroiditis and rheumatoid arthritis; one of the potential causes is the proinflammatory state in GPS and the autoantigen upregulation, which may lead to autoantibody generation. We noted a borderline low C3, which could be related to a potential autoimmune disorder, but further investigations for possible autoimmune disorders were not conducted due to loss of follow-up [16].

## 4. Conclusion

We highlight a case of GPS with hepatomegaly and atypical hematologic abnormalities, including low lymphocytes and monocytes with normal neutrophils, further supporting the novel studies on the effect of NBEAL2 mutation that suggest it does not only affect platelets but also many other blood cell precursors, potentially contributing to the association with the high susceptibility to infections and the high prevalence of autoimmune disorders in GPS patients.

We also highlight the need for a guideline for the screening of autoimmune disorders in patients with GPS.

## Figures and Tables

**Table 1:** Laboratory tests on admission.

Test	Result	Normal range
White cell count (WBC)	3.62 K/μL	4.5–10 K/μL
Absolute neutrophil (ANC)	2870	1500–8000
Neutrophil %	79.47%	41%–76%
Lymphocyte %	16.4%	20%–41%
Monocyte %	3.19%	3%–13%
Eosinophil %	0.74%	0%–6%
Basophil %	0.2%	0%–2%
Red cell count (CBC)	5.38 M/μL	4.3–5.3 M/μL
Hemoglobin (HGB)	12.9 g/dL	∼11.5–15.5 g/dL (adolescents)
Hematocrit (HCT)	38.9%	33%–44%
Mean corpuscular volume (MCV)	74.2 fL	77–87 fL
Platelet (PLT)	59 × 10^9^/L	140–450 × 10^9^/L
Prothrombin time (PT)	16.5 s	∼11–15 s
PTT (partial thromboplastin time)	28.6 s	25–35 s
INR	1.28	0.8–1.2
Fibrinogen	192 mg/dL	200–400 mg/dL
D-dimer	0.47 μg/mL	0–0.5 μg/mL
ESR (erythrocyte sed. rate)	3 mm/hr	0–20 mm/hr
CRP (C-reactive protein)	0.6 mg/dL	0–1 mg/dL
BUN (blood urea nitrogen)	12.2 mg/dL	7–20 mg/dL
Creatinine	0.45 mg/dL	0.5–1.2 mg/dL (age-dependent)
Sodium (Na)	139 mEq/L	135–145 mEq/L
Potassium (K)	4.9 mEq/L	3.5–5.1 mEq/L
Calcium (Ca)	9.6 mg/dL	8.5–10.5 mg/dL
Chloride (Cl)	105 mEq/L	98–107 mEq/L
Phosphorus (PO_4_)	4.4 mg/dL	2.5–4.5 mg/dL
Ferritin	32 ng/mL	15–200 ng/mL
LDH (lactate dehydrogenase)	192 U/L	140–280 U/L
ALT (alanine transaminase)	10 U/L	7–56 U/L
AST (aspartate transaminase)	16.8 U/L	10–40 U/L
Glucose	103 mg/dL	70–110 mg/dL (fasting)
Triglycerides	53 mg/dL	< 150 mg/dL
Uric acid	2.8 mg/dL	2.0–7.0 mg/dL

**Table 2:** Differential diagnosis of disorders with a similar presentation to GPS.

Diagnosis	CBC	Labs	Smear	Bone marrow	Symptoms	Ref.
Gray platelet syndrome	Thrombocytopenia, large platelets (MPV)	NBEAL2 mutation	Large, gray platelets lacking alpha-granules	Normal or slightly increased megakaryocytes	Mild/moderate bleeding, bruising, splenomegaly	[3]
GFI1B-related thrombocytopenia	Isolated thrombocytopenia	GFI1B mutation	Large, hypogranular platelets with dysplastic features	Dysmegakaryopoiesis	Bleeding tendency with variable severity	[7]
GATA1-related thrombocytopenia	Thrombocytopenia ± anemia	GATA1 mutation	Large platelets with abnormal morphology	Dyserythropoiesis ± dysmegakaryopoiesis	Bleeding, anemia	[8]
Thrombocytopenia, anemia and myelofibrosis (G6b-B–related thrombocytopenia)	Thrombocytopenia	MPIG6B mutation	Large platelets	Megakaryocyte abnormalities	Severe bleeding	[9]
Immune thrombocytopenic purpura (ITP)	Isolated thrombocytopenia	Elevated platelet-associated antibodies; normal inflammatory markers.	Normal platelet morphology	Increased megakaryocytes	Bruising, petechiae, mucosal bleeding	[10]
Hemophagocytic lymphohistiocytosis (HLH)	Pancytopenia or thrombocytopenia	↑ Ferritin, triglycerides, IL-2 receptors ↓Fibrinogen	Pancytopenia	Hemophagocytosis: macrophages engulfing blood cells	High fever, hepatosplenomegaly, fatigue	[11]
Bernard–Soulier syndrome (BSS)	Thrombocytopenia, large platelets (MPV)	Abnormal ristocetin cofactor assay with normal platelet aggregation to other agonists	Giant platelets, normal alpha-granules	Normal or slightly increased megakaryocytes	Moderate/severe bleeding	[12]
Myelofibrosis (MF)	Thrombocytopenia (advanced stages)	JAK2/MPL/CALR mutations; leukoerythroblastic features	Teardrop cells, abnormal platelets	Hypercellular marrow, and fibrosis in advanced stages	Fatigue, splenomegaly, hepatomegaly	[13]
Von Willebrand disease Type 2B	Normal platelet count or mild thrombocytopenia	↓VWF, ↑ristocetin-induced aggregation	Normal or small platelets	Normal	Bleeding tendency	[14]

## Data Availability

No datasets were generated or analyzed for this case report.
